# Risk factors for early TB treatment interruption among newly diagnosed patients in Malaysia

**DOI:** 10.1038/s41598-021-04742-2

**Published:** 2022-01-14

**Authors:** Qudsiah Suliman, Poh Ying Lim, Salmiah Md. Said, Kit-Aun Tan, Nor Afiah Mohd. Zulkefli

**Affiliations:** 1 Department of Community Health, Faculty of Medicine and Health Sciences, Universiti Putra Malaysia, UPM, 43400 Serdang, Selangor Malaysia; 2 Ministry of Health, Putrajaya, Wilayah Persekutuan Putrajaya, Malaysia; 3 Department of Psychiatry, Faculty of Medicine and Health Sciences, Universiti Putra Malaysia, UPM, 43400 Serdang, Selangor Malaysia

**Keywords:** Infectious diseases, Tuberculosis

## Abstract

TB treatment interruption has resulted in delayed sputum conversion, drug resistance, and a high mortality rate and a prolonged treatment course, hence leading to economic and psychosocial affliction. To date, there are limited studies investigating the physico-social risk factors for early treatment interruptions. This prospective multicenter cohort study aimed to investigate the risk factors for early treatment interruption among new pulmonary tuberculosis (TB) smear-positive patients in Selangor, Malaysia. A total of 439 participants were recruited from 39 public treatment centres, 2018–2019. Multivariate Cox proportional hazard analyses were performed to analyse the risk factors for early treatment interruption. Of 439 participants, 104 (23.7%) had early treatment interruption, with 67.3% of early treatment interruption occurring in the first month of treatment. Being a current smoker and having a history of hospitalization, internalized stigma, low TB symptoms score, and waiting time spent at Directly Observed Treatment, Short-course centre were risk factors for early treatment interruption. An appropriate treatment adherence strategy is suggested to prioritize the high-risk group with high early treatment interruption. Efforts to quit smoking cessation programs and to promote stigma reduction interventions are crucial to reduce the probability of early treatment interruption.

## Introduction

TB treatment interruption will bring significant impacts to patients, including delayed sputum conversion, drug resistance^1,2^, prolonged infectiousness in the community, high mortality rate^3,4^, and prolonged treatment course, hence leading to economic and psychosocial affliction^5,6^. Malaysia is an intermediate to high TB burden country that has depicted an increasing trend of TB mortality (per 100,000 population) from 5.33 in 2014 to 6.55 in 2018. Selangor, the most populous and urbanized state in Malaysia, has faced challenges in implementing TB control strategies. From 2014 to 2018, the TB treatment interruption rate in Selangor was far above the predetermined target (2%), and the latest was 10.9% in 2018. In parallel, Malaysia had a suboptimal TB treatment success rate, ranging from 78 to 84% between the years 2013 and 2016, compared to the WHO's target level of 90%.

Understanding the time to early treatment interruption during the intensive phase of TB (or early treatment interruption) is crucial to tailor the time-relevance adherence strategy. Poor compliance during the intensive phase was found to contribute to unfavorable treatment outcomes and a high risk of mortality, thus raising a priority need to retrieve non-adherent patients during the intensive phase^3,7^. Based on previous longitudinal studies, risk factors for treatment interruption included having a previous history of TB diagnosis^8,9^, travel distance^8,10^, and HIV disease^8,9,11,12^. Of elicits, the lack of assessment for cognitive and psychosocial determinants may obscure the anticipation of social risk profiling in TB management^13–15^.

As one of the social determinants, TB stigma contributes to a detrimental effect on TB control via the sentiment of disgrace and blame, hence the internalization of the community’s skeptical judgments that lead to the abandon treatment in the DOTS program^16^. DOTS program is core policy, fee exemption policy for anti-TB drug in developing countries and fixed-dose combination tablets for TB treatment^17,18^. Likewise, the defaulter tracing and retrieval system is distinctly outlined through National Tuberculosis Control Program in Malaysia. Despite of free TB treatment to all patients, early treatment interruption is remained a problem in Malaysia, thus this study hypothesised psychological factors such as internal stigma has a potential role in this problem. Therefore, this study aims to identify the time to early TB treatment interruption and risk factors for early treatment interruption among new pulmonary TB smear-positive patients in urban districts of Selangor.

## Materials and methods

Methodological details of the present study have been published elsewhere^19^. Hence, only short descriptions pertaining to the study settings, design, and procedure are explained in the following sections. The study protocol and methods of obtaining consent were approved by the National Medical Research and Ethics Committee (NMREC) of the National Institute of Health, Ministry of Health Malaysia (Reference number: NMRR-18-1635-42371) and the Ethics Committee for Research Involving Human Subject Universiti Putra Malaysia (JKEUPM). All the methods were performed in accordance with the relevant guidelines and regulations.

### Study setting, design and procedure

The present multicentric prospective cohort study was conducted at five public hospitals and 34 health clinics, which has DOTS center providing the treatment to TB patients in the urban districts (Petaling, Hulu Langat, Gombak, Klang and Sepang) of Selangor. All new pulmonary TB smear-positive patients who started treatment from November 2018 to August 2019 were consecutively enrolled in the present study. The outcome of interest was early treatment interruption during intensive phase treatment. It is defined as early treatment interruption for 14 days or more, or loss to follow up according the management of Tuberculosis-Clinical Practice Guidelines. Intensive phase is defined as standard TB treatment, on a daily basis during the first 60 days of initiating TB treatment (completion of 60 doses). For selective cases, the intensive phase may be prolonged depending on treatment response, missing follow up or sputum conversion.

Inclusion criteria were being Malaysian, aged 18 years and above, able to understand Malay or Chinese (Mandarin) language, and mentally capable. Patients who had their diagnosis changed to non-TB diagnosis, multi-drug resistant TB patients and started TB treatment at private facilities, were excluded from the study. Severely ill (bedridden patients), pre-existing mental illness on treatment illness on treatment or suggestive of depressive symptoms based on Patient Health Questionnaire (PHQ-9) screening were be excluded.

Data extraction from medical records and tuberculosis information system (TBIS) forms comprising 32 formats on recording and reporting TB treatment history and outcome was performed by five trained enumerators.

At baseline, a self-administered questionnaire was completed by participants to elicit information on sociodemographic factors, risky behavior (smoking status, history of substance abuse and alcohol consumption), symptom assessments (frequency of symptom including dry cough, cough with sputum, cough up blood, loss of weight, fever, night sweat and loss of appetite for past one week), traditional or alternative treatment and health service factors information (18 items on knowledge on TB treatment), motivation factors (health belief, social support and internalized stigma), behavioral skills (cue to action and self-efficacy) and health services (travel distance and waiting time). Meanwhile, PROFORMA was applied to elicit information on clinical characteristics (comorbidities, chest X-ray grading, baseline body weight, and history of hospitalization) and treatment status. The sample size was calculated as per the time-to-event data formula^20^. Using a desired hazard ratio (HR) of 3.0 for the travel distance factor^8^ and 0.20 for the hypothesized proportion of early treatment interruption, the required sample size was at least 438 to achieve a 5% significant level at 80% power.

As outlined in the National TB Control Policy, all TB patients are subjected to DOTS policy, which require patient to come to treatment centre for daily basis in order to observe on pill takings. Follow up via calling patients or next of kin if patient is absent in their treatment to ensure they will continue their treatment.

### Statistical analysis

This outcome was binary, with one (1) corresponding to when the subject had an event (early treatment interruption) and zero (0) corresponding to when patients did not experience the event (i.e., early treatment interruption) at day 60 or until last date of TB treatment (completion of 60 doses), died regardless of the cause, or moved away or transferred to other treatment centres outside the study location.

Cox proportional hazards (PH) regression was computed to identify the risk factors for early treatment interruption using IBM SPSS Version 25.0^21^. Median of survival time which defined at middle value of survival time was demonstrated as survival probability was not reached 50% by end of study. The Kaplan–Meier technique was used to obtain the survival estimate and to plot the survival distribution. Study variables with a *p* value ≤ 0.25 in univariate Cox PH regression were tested in multivariate Cox PH regression analysis with the backward elimination method. Only study variables with *p* < 0.05 were retained in the final model. The results are reported as hazard ratios (HRs) and their 95% confidence intervals (CI). The assumption of proportionality of hazards and model fit were checked.

### Ethics approval and consent to participate

The study protocol and methods of obtaining consent were approved by the National Medical Research and Ethics Committee (NMREC) of the National Institute of Health, Ministry of Health Malaysia (Reference number: NMRR-18-1635-42371) and the Ethics Committee for Research Involving Human Subject Universiti Putra Malaysia (JKEUPM). All the methods were performed in accordance with the relevant guidelines and regulations. Written informed consent was obtained from participants. Additional information sheets to further explain the study objectives and methods were distributed. Confidentiality of personal information and survey responses were maintained in the strictest confidence by keeping the identifiable information anonymous during data analysis. Throughout the follow-ups, the defaulter tracing system was ensured by all levels of care.

## Results

### Characteristics of study participants

**Figure 1:**
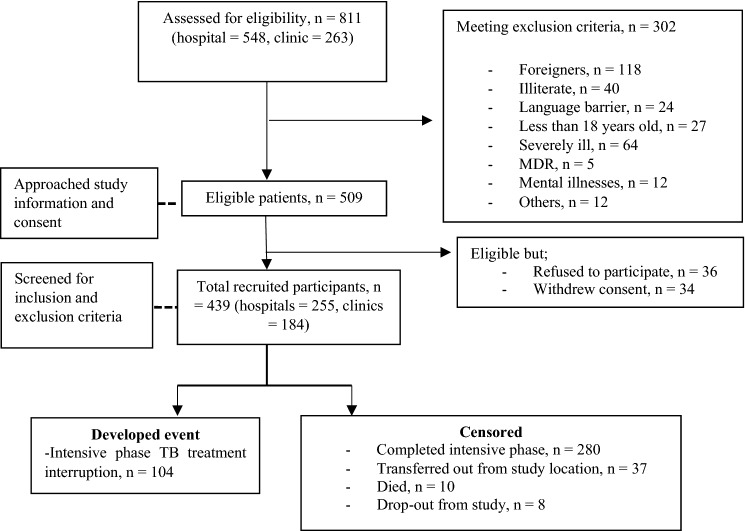
Study profile for the assessment of early treatment interruption among pulmonary TB smear-positive patients in urban districts, Selangor.

The study profile is depicted in Fig. 1. During the recruitment period, 509 eligible patients were approached and assessed. Of these, 70 patients refused to participate. As such, the respond rate was 86.2%.

The present sample was predominantly male (65.5%), Malay (75.2%), married (52.8%), and of hospital settings (51.5%). The median family income was RM1959.50 [(IQR = Interquartile Range = 25th percentile, 75th percentile) = 1200, 3000], whereas the mean ± SD age was 41.82 ± 15.20 years. In the present sample, 34.6% were current smokers, 32.8% were diabetes mellitus patients, and 5% were HIV-positive patients. Approximately 51% of the participants had a diagnosis of grade 3 (moderately advanced) or grade 4 (far advanced) chest X-ray findings, whereas 33.7% had practiced at least one type of alternative or traditional medicine for TB disease.

### Duration of time to early treatment interruption among pulmonary tb smear positive patients

**Figure 2:**
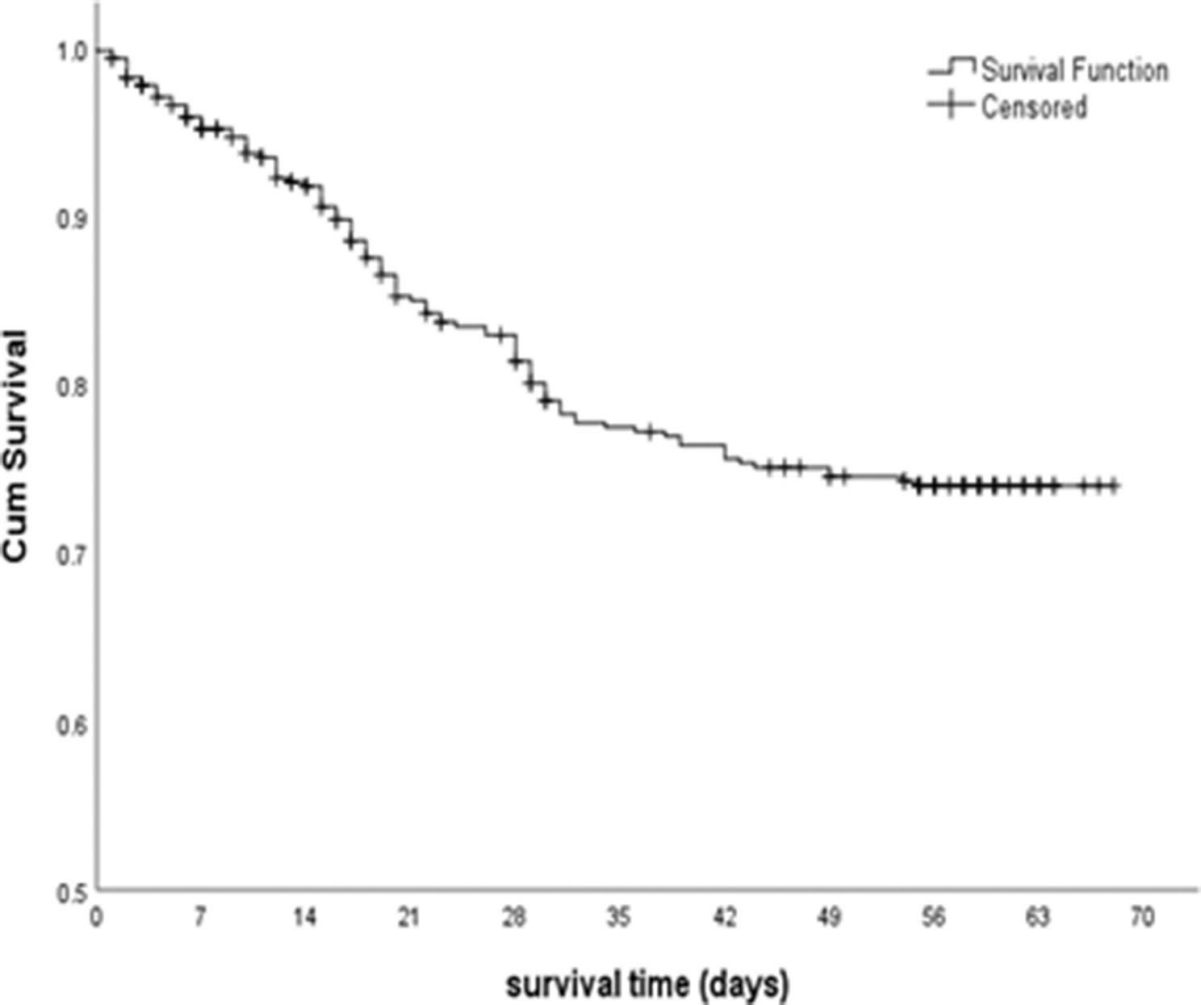
Survival distribution of early treatment interruption among new pulmonary TB smear-positive patients in urban districts, Selangor (n = 439).

Of 439 participants, the early treatment interruption rate was 23.7% (n = 104). The median time to early treatment interruption was 56.00 days (95% CI 55.00–56.00). The life table and the participants’ survival throughout the treatment course are demonstrated in Table 1 and Fig. 2, respectively. There was a rapid decrease in the survival curve, particularly in the first 28 days of TB treatment, but it reached a plateau after day 42. The proportion of events was highest in the interval of Day 15–28. Approximately 67.3% of early TB interruptions occurred during the first month of treatment. The overall survival probability at the end of early treatment interruption was 73.9%. There were 8.4% (n = 37) of participants transferred out from study location, 2.3% (n = 10) of participants had died whilst 1.8% (n = 8) of participants opted to drop out from study follow up.

**Table 1:** Life table of early treatment interruption among newly diagnosed pulmonary TB smear-positive patients in urban districts, Selangor (n = 439).

Time interval (days)	Number of entering interval	Number of events*	Number of dropped out	Proportion of event*	Cumulative proportion surviving at end of interval
0–14	439	34	32	0.08	0.920
15–28	373	36	16	0.10	0.829
29–42	321	25	7	0.08	0.764
43–56	289	9	16	0.03	0.739
> 56	264	0	264	0	0.739

*Event: early treatment interruption.

### Risk factors of early treatment interruption

Univariate Cox PH regression between early treatment interruption and study variables are shown in Tables 2, 3, and 4. Among sociodemographic factors and high risk behaviours, ethnicity, marital status, education levels and smoking status were associated with risk of early treatment interruption (Table 2). Regarding clinical characteristics (Table 3) and information, motivation, behavioural skills and health service factors (Table 4), history of hospitalization upon starting treatment, knowledge, social support, internalized stigma, waiting time at DOTS centre were significant factors of risk of early treatment interruption. Study variables with a *p* value ≤ 0.25 were tested in a multivariate Cox model. Based on the final multivariate Cox model, being a current smoker (HR = 1.649, 95% CI 1.107–2.458, *p* = 0.014), having a history of hospitalization upon starting treatment (HR = 2.539, 95% CI 1.648–3.910, *p* < 0.001), low TB symptoms score (HR = 0.954, 95% CI 0.912–0.998, *p* = 0.041), internalized stigma (HR = 1.056, 95% CI 1.018–1.096, *p* = 0.004), and waiting time at DOTS centre (HR = 1.005, 95% CI 1.001–1.009, *p* = 0.042) were associated with early treatment interruption (Table 5).

**Table 2:** Risk of early treatment interruption by sociodemographic factors and high-risk behaviours (n = 439).

Variables	Early treatment interruption			Univariate Cox PH analysis	
	Yes	No	Total	Crude HR (95% CI)	*p* value
**Age**					
< 60	92 (24.4)	285 (75.6)	377 (85.9)	Reference	
≥ 60	12 (19.3)	50 (80.7)	62 (14.1)	0.836 (0.458–1.526)	0.559
**Family income**^**a**^	RM1959.50 (RM1800.00)			1.000 (1.000–1.000)	0.227
**Number of household [Median (IQR)]**	4 (*3,6*)			0.975 (0.897–1.060)	0.549
**Gender**					
Male	76 (26.0)	216 (74.0)	292 (66.5)	1.455 (0.943–2.244)	0.09
Female	28 (19.0)	119 (81.0)	147 (33.5)	Reference	
**Ethnicity**					
Malay	66 (20.0)	264 (80.0)	330 (75.2)	Reference	
Non-Malay	38 (35.0)	71 (65.0)	109 (24.8)	1.886 (1.265–2.812)	0.002*
**Marital status**					
Married	57 (24.6)	175 (75.4)	232 (52.8)	Reference	
Single	34 (21.0)	127 (79.0)	161 (36.7)	0.920 (0.602–1.407)	0.701
Widowed	3 (12.5)	21 (87.5)	24 (5.5)	0.470 (0.147–1.502)	0.203
Divorced	7 (46.7)	8 (53.3)	15 (3.4)	2.668 (1.216–5.854)	0.014*
Separated	3 (42.9)	4 (57.1)	7 (1.6)	1.675 (0.525–5.349)	0.384
**Educational level**					
No formal education	4 (21.1)	15 (78.9)	19 (4.3)	1.486 (0.503–4.391)	0.474
Primary school	24 (39.3)	37 (60.7)	61 (13.9)	3.053 (1.657–5.628)	< 0.001*
Secondary school	58 (25.0)	174 (75.0)	232 (52.8)	1.841 (1.122–3.312)	0.024*
Tertiary education	18 (14.2)	109 (85.8)	127 (28.9)	Reference	
**Employment status**					
Employed	31 (22.5)	107 (77.5)	267 (60.8)	0.907 (0.592–1.388)	0.653
Unemployed	67 (25.1)	200 (74.9)	138 (31.4)	0.787 (0.341–1.814)	0.574
Retiree	6 (17.6)	28 (82.4)	34 (7.7)	Reference	
**Staying status**					
Nuclear family	59 (24.5)	182 (75.5)	241 (54.9 )	Reference	
Extended family	11 (18.3)	49 (81.7)	60 (13.7)	0.767 (0.403–1.460)	0.420
Single parent family	7 (18.9)	30 (81.1)	37 (8.4)	0.770 (0.352–1.686)	0.513
Staying with other relatives	2 (50.0)	2 (50.0)	4 (0.9)	3.878 (0.94515.920)	0.060
Staying with friend	9 (30.0)	21 (70.0)	30 (6.8)	1.542 (0.764–3.110)	0.227
Staying alone	10 (23.8)	32 (76.2)	42 (9.6)	1.051 (5.370–2.054)	0.885
Staying with others	6 (24.0)	19 (76.0)	25 (5.7)	1.074 (0.464–2.488)	0.868
***High risk behaviour***					
**Smoking status**					
Non-smoker	54 (18.8)	233 (81.2)	152 (34.6)	Reference	
Current smoker	50 (32.9)	102 (67.1)	287 (65.4)	2.005 (1.364–2.946)	< 0.001*
**Alcohol consumption**					
No	93 (23.0)	311 (77.0)	35 (8.0)	Reference	
Yes	11 (31.4)	24 (68.6)	404 (92.0)	1.715 (0.917–3.207)	0.091
**Illicit drug intake**					
No	95 (23.3)	313 (76.7)	31 (7.1)	Reference	
Yes	9 (29.0)	22 (71.0)	408 (92.9)	1.434 (0.723–2.841)	0.302

*p < 0.05.

^a^1USD≈RM4.20, mean family income was considered to be within B40 group (low income group).

**Table 3:** Risk of early treatment interruption by clinical characteristics (n = 439).

Variables	Early treatment interruption			Univariate Cox PH analysis	
	Yes	No	Total	Crude HR (95% CI)	*p* value
**Comorbid factors**					
**Diabetes mellitus**					
No	72 (24.4)	223 (75.6)	144 (32.8)	Reference	
Yes	32 (15.3)	112 (84.7)	295 (67.2)	0.884 (0.583–1.340)	0.560
**Chronic obstructive pulmonary disease**					
No	101 (23.3)	332 (76.7)	6 (1.4)	Reference	
Yes	3 (50.0)	3 (50.0)	433 (98.6)	2.188 (0.694–6.901)	0.181
**Chronic liver disease**					
No	100 (23.2)	331 (76.8)	8 (1.8)	Reference	
Yes	4 (50.0)	4 (50.0)	431 (98.2)	2.389 (0.878–6.494)	0.088
**Chronic renal failure**					
**No**	102 (23.9)	324 (76.1)	13 (3.0)	Reference	
Yes	2 (15.4)	11 (84.6)	426 (97.0)	0.611 (0.151–2.475)	0.490
**HIV/AIDS status**					
Negative	98 (23.5)	319 (76.5)	22 (5.0)	Reference	
Positive	6 (27.3)	16 (72.7)	417 (95.0)	0.825 (0.362–1.883)	0.648
**Underlying comorbid**					
No	56 (22.9)	188 (77.1)	195 (44.4)	Reference	
Yes	48 (24.6)	147 (75.4)	244 (55.6)	1.066 (0.725–1.567)	0.747
**TB disease related factors**					
**TB symptoms score [Mean (SD)]**	14.55 (4.35)			0.961 (0.919–1.006)	0.086
**Body weight [Median(IQR)]**	52.50 (43,62)			0.998 (0.985–1.011)	0.721
**Chest X-ray grading**					
Grade 1(no lesion)	6 (25.0)	18 (75.0)	24 (5.5)	1.320 (0.444–3.929)	0.618
Grade 2 (minimal lesion)	38 (19.9)	153 (80.1)	191 (43.5)	1.038 (0.463–2.324)	0.928
Grade 3 (moderately advanced)	53 (28.6)	132 (71.4)	185 (42.1)	1.460 (0.604–0.321)	0.402
Grade 4 (far advanced)	7 (17.9)	32 (82.1)	39 (8.9)	Reference	
**TB treatment related disease**					
**Alternative/medicine practice**					
No	75 (25.7)	216 (74.3)	148 (33.7)	Reference	
Yes	29 (19.6)	119 (80.4)	291 (66.3)	0.813 (0.530–1.248)	0.344
**History of hospitalization upon starting treatment**					
No	30 (14.1)	183 (85.9)	213 (48.5)	Reference	
Yes	74 (32.7)	152 (67.3)	226 (51.5)	2.874 (1.880–4.396)	0.001*

*HR* Hazard Ratio, *CI* Confidence Interval.

*p < 0.05.

**Table 4:** Risk of early treatment interruption by information, motivation, behavioural skills and health service factors (n = 439).

Variable	Mean (SD)	Minimum/maximum score	Crude HR (95% CI)	*p* value
***Informational factor***				
**Knowledge**	10.78 (3.72)	0.00/18.00	0.948 (0.903–0.996)	0.035*
***Motivational factors***				
**Health belief**				
Perceived susceptibility	15.46 (2.44)	5.00/20.00	0.967 (0.897–1.043)	0.380
Perceived severity	16.06 (2.40)	5.00/20.00	0.970 (0.897–1.048)	0.442
Perceived barrier	36.92 (6.50)	18.00/64.00	1.028 (0.898–1.037)	0.059
Perceived benefit	16.10 (2.62)	5.00/20.00	0.965 (0.898–1.037)	0.333
Social support	33.61 (5.79)	13.00/48.00	0.957 (0.925–0.990)	0.011*
Internalized stigma	24.57 (4.84)	10.00/40.00	1.063 (1.025–1.103)	0.001*
***Behavioural skills factors***				
Cue to action	31.61 (5.27)	10.00/40.00	0.976 (0.941–1.011)	0.180
Self-efficacy	57.97 (9.00)	16.00/80.00	0.991 (0.971–1.011)	0.374
***Health services factors***				
Travel distance to DOTS centre	8.00 (3.00, 13.00)^a^	1.00/85.00	1.005 (0.990–1.020)	0.544
Travel distance to follow up centre	8.00 (3.00, 13.00)^a^	1.00/120.00	1.005 (0.991–1.019)	0.516
Waiting time at DOTS centre	30.00 (10.00,50.00)^a^	5.00/180.00	1.006 (1.001–1.010)	0.008*
Waiting time at follow up centre	60.00 (30.00,90.00)^a^	5.00/240.00	1.001 (0.998–1.004)	0.518

*HR* Hazard Ratio, *CI* Confidence Interval, *SD* Standard deviation.

*p < 0.05.

^a^median (IQR).

**Table 5:** Risk factors for early treatment interruption among pulmonary TB smear-positive patients in urban districts, Selangor (n = 439).

Characteristics	Adjusted HR	SE	95% CI	*p-*value
**Smoking status**				
Non-smoker	Reference			
Current smoker	1.649	0.203	1.107–2.458	0.014*
**History of hospitalization upon starting treatment**				
No	Reference			
Yes	2.539	0.220	1.648–3.910	< 0.001*
**Baseline TB symptoms score**	0.954	0.023	0.912–0.998	0.041*
**Baseline internalized stigma**	1.056	0.019	1.018–1.096	0.004*
**Waiting time spent at DOTS centre**	1.005	0.002	1.001–1.009	0.042*

Using the backward LR method, Cox PH regression – χ^2^ = 53.50, df = 5, *p* < 0.001, PH assumptions were tested using correlation tests between partial residuals and survival time rank (nonsignificant correlations, *p* > 0.05 were reported), and no issue of multicollinearity was detected.

*HR* Hazard Ratio, *SE* Standard Error, *CI* Confidence Interval.

*p < 0.05.

## Discussion

This study elucidated the understanding of time to early treatment interruption and its risk factors. The early TB interruption rate yielded in the present study surpassed the national target of 2% and recent state surveillance data of 10.9% in 2018. This study concurs with the latter studies in terms of predominantly hospitalized TB patients among the recruited participants. One of the possible reasons is that the majority of respondents were from the low median family income group. Poor urban groups were found to exhibit low socioeconomic status, inadequate social assistance, vulnerable socioeconomic profiles, and thus poor access to the health care system^22,23^. The high early treatment interruption rate reported in this study therefore heightened the need for prompt intervention and policies that should be strategized systematically according to gathered evidence.

The significant effects of smoking status on early TB treatment interruption endorsed a previous local finding in northern Malaysia reporting that current smokers had a three times higher risk of treatment interruption than noncurrent smokers^24^. As the double burden of TB and smoking are prevalent in Selangor state, this finding should further inform the development of quit smoking program among TB patients.

The present study depicted that TB symptoms were negatively associated with early treatment interruption^25–27^. Experiencing more symptoms leads to fear that nurtures susceptibility and severity and hence motivations to persevere with treatment, suggested by a previous study which demonstrated that severity reduced the chances of treatment interruption among multi- and extensively drug-resistant tuberculosis patients^28^. It appears that sputum conversion and treatment response to TB treatment are frequently expedited in patients with mild symptoms, which in turn induce the sensation of being cured, resulting in early dropout^29^. This, however, warrants further evaluations to explore the patterns of discontinuation of treatment among those with mild symptoms upon diagnosis.

It is surprising that patients with a history of hospitalization upon starting treatment had a higher risk of early treatment interruption. In Malaysia, some hospitalized TB patients would have subsequent DOTS monitoring at health clinics or other treatment centres, where this will increase the chances of patients not continuing their treatment. In-ward management should also have the capacity of a dedicated managing team to ensure that all recordings and reports for discharge or interfacility referrals are properly documented and communicated across the level of care. Future study is suggested to understand the duration of hospitalization, condition of patients and causes of hospitalization might possibly affect the risk of early treatment interruption. Hospitalization factor could be investigated further by comparing the early interruption treatment between hospitalized patients and non-hospitalized patients in future study.

Internalized stigma was a risk factor for early treatment interruption^30,31^, thus providing new empirical evidence from the local perspective. Some TB patients fear that other people see them as HIV/AIDS patients^32,33^. Other TB patients experience the suppression of self-esteem secondary to stigmatization, contributing to treatment avoidance^34,35^. The foregoing postulation warrants further evaluation of public stigma such as negative attitudes, beliefs, and behaviors among the local community by clarifying the different drivers and dimensions of TB stigma to endeavour the framework of the stigma reduction strategy.

Health system matters, such as longer waiting time spent by participants at the DOTS centre, increase the risk of early treatment interruption^36,37^. The experience of long wait is burdensome for patients, in the sense of frequent medical leave from their jobs, and impairs their income for living expenses, thus compromising the consistency of DOTS monitoring^38^. In addition, a longer waiting time undermines the patients’ satisfaction with health system delivery, resulting in a high drop out from DOTS^39^. Therefore, the flow management of DOTS monitoring should be refined to optimize the capacity of providers and efficient process flow by taking the waiting time into consideration.

In essence, the present study provides local updates beyond the biomedical attributes extracted from medical records or disease registries. One of the strengths of this study was median time of early treatment interruption was identified and further investigate its associated factors, using survival analysis. Censored data (16.9%) including participants drop out from study, transferred out from study and passed away still retained in the analysis, thus remaining the power of analysis and also reducing the selection bias. Life table could explain further the probability of early treatment interruption, controlling by number of dropped out. Understanding the barriers of participants drop out or transferred out from the study is suggested as this could reduce the probability of early treatment interruption. The present study also offers a better understanding and simpler explanation of early treatment interruption in light of psychosocial influence via the information, motivation and behavior skills (IMB) model framework. Therefore, the present findings may benefit organizations and policy makers in designing time-relevant, theory-based adherence strategies in TB case holding and management. Above all, the present study was conducted in public health centre settings, thus limiting its generalizability to other study populations. Investigating the early treatment interruption characteristics as exposure variable with the treatment outcome (sputum conversion) could further understand the role of early interruption treatment on treatment outcome, eventually could convince the policy markers to design appropriate the program and strategies to reduce the chances of early interruption treatment.

## Conclusions

The present findings suggest that the early TB treatment interruption among new pulmonary TB smear-positive patients in urban districts was high and was dictated by smoking status, history of hospitalization upon starting treatment, lower TB symptom score, internalized stigma, and longer waiting time at the DOTS centre. As informed by these findings, the strategies towards improving TB treatment adherence and TB treatment outcome in urban district Selangor should target high-risk groups endeavoring stigma reduction strategy and quitting smoking intervention as well as improving waiting time at the DOTS centre. It is also hoped that future studies could not only assess more study domains by catering objective assessments of health services but also incorporate various drivers and dimensions of TB stigma, such as public or organizational stigma.

## Data Availability

The datasets analyzed during the current study are available in the Mendeley data, http://dx.doi.org/10.17632/pc773fty8m.1.

## Abbreviations

TB: Tuberculosis
DOTS: Directly Observed Treatment, Short-course
PH: Proportional hazard
HR: Hazard ratio
SD: Standard deviation
IQR: Interquartile range (25th percentile, 75th percentile)
CI: Confidence interval
